# Expression of human growth hormone in the milk of transgenic rabbits with transgene mapped to the telomere region of chromosome 7q

**DOI:** 10.1007/s13353-012-0110-4

**Published:** 2012-08-17

**Authors:** Daniel Lipinski, Joanna Zeyland, Marlena Szalata, Andrzej Plawski, Malgorzata Jarmuz, Jacek Jura, Aleksandra Korcz, Zdzislaw Smorag, Marek Pienkowski, Ryszard Slomski

**Affiliations:** 1 Department of Biochemistry and Biotechnology, Poznan University of Life Sciences, Dojazd 11, 60-632 Poznan, Poland; 2 Institute of Human Genetics, Polish Academy of Sciences, Strzeszynska 32, 60-479 Poznan, Poland; 3 Department of Animal Reproduction, National Research Institute of Animal Production, 32-083 Balice, Poland; 4 Pien Gen Biomedical Corporation, Knoxville, TN USA

**Keywords:** Whey acidic protein promoter, Recombinant human growth hormone, Transgenic animals

## Abstract

The advent of transgenic technology has provided methods for the production of pharmaceuticals by the isolation of these proteins from transgenic animals. The mammary gland has been focused on as a bioreactor, since milk is easily collected from lactating animals and protein production can be expressed at very high levels, including hormones and enzymes. We demonstrate here the expression pattern of recombinant human growth hormone (rhGH) in transgenic rabbits carrying hGH genomic sequences driven by the rat whey acidic protein (WAP) promoter. The transgene was mapped to the q26-27 telomere region of chromosome 7q by fluorescence in situ hybridization (FISH). Nearly 30 % of the F1 generation demonstrated the presence of transgene. The recombinant growth hormone was detected in the milk of the transgenic rabbit females, but not in serum, up to the level of 10 μg/ml. Ectopic expression of the transgene in the brain, heart, kidney, liver, and salivary gland was not observed, indicating that a short sequence of rat WAP promoter (969 bp) contained essential sequences directing expression exclusively to the mammary gland. The biological activity of recombinant growth hormone was measured by immunoreactivity and the capability to stimulate growth of the hormone-dependent Nb211 cell line.

## Introduction

Growth hormone (GH) is a factor controlling growth in the vertebrates and metabolism of mammals by the stimulation of a protein synthesis and a lipid degradation. Human GH (hGH) is composed of a single polypeptide chain of 191 amino acids with a molecular mass of 22 kDa. It contains two internal disulfide bonds and a tertiary structure that includes four alpha helices arranged in an antiparallel fashion. The genomic sequence of hGH encompasses 1,632, nucleotides including five exons. The growth hormone gene cluster is assigned to chromosome 17q23-q24 (Niall et al. 1971; Owerbach et al. 1980; Xu et al. 1988; Lipiński et al. 2003).

Growth hormone deficiencies (GHD) in humans are primarily due to pituitary disease, adenoma, and trauma, as well as primary deficiencies. The clinical manifestation of human growth hormone deficiencies in adults results in severe cardiovascular disease, causing premature death, increased fat mass, reduced muscle mass, lower cardiac output, lower bone density, and increased protein lipids, leading to a substantial decrease of life quality in affected individuals. In children, growth hormone deficiencies additionally result in severe growth retardation and can be congenital (e.g., Turner’s syndrome) or acquired due to tumors or renal insufficiencies (Collett-Solberg 2011). Reduced bone mineral density (BMD) had been reported in patients with isolated GHD or with multiple pituitary hormone deficiencies (Colao et al. 1999). The only known effective treatment of this and related conditions is human growth hormone replacement therapy. In addition, a number of other medical conditions, including Crohn’s disease, catabolic conditions like burns, coronary vascular disease, premature aging, idiopathic osteoporosis, and many others, have been demonstrated to be successfully treated by human growth hormone replacement therapy (Ayyar 2011). The first use of growth hormone therapy for GH deficiency was reported in 1958 (Raben 1958). Originally, hGH therapeutic preparations were derived from human cadaver pituitaries, but in the mid-1980s, such production was linked to a risk for Creutzfeldt–Jakob disease (CJD) and was terminated (Hintz 1995). This situation started to stimulate the development of new strategies and revealed the market of a recombinant human GH (rhGH) beginning in 1985 (Kemp and Frindik 2011).

In the present study, we describe an expression of a recombinant human growth hormone in the mammary glands of transgenic rabbits at the transcription and the translation levels, a stability of the transgene transmission, and a mapping of the transgene by fluorescence in situ hybridization (FISH). The recombinant WAP:6xHishGH vector containing the hGH gene used in this study was sufficient to target rhGH expression into the mammary gland of lactating animals.

## Materials and methods

### Sample collection

**Scheme 1:**
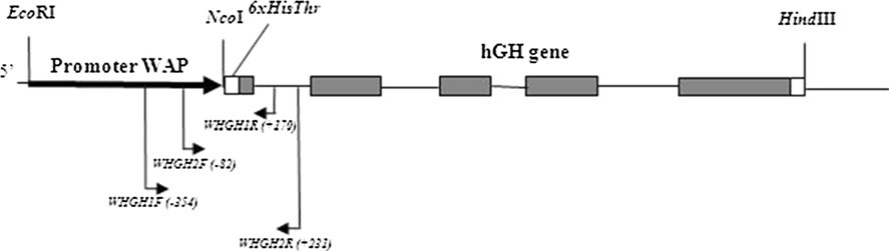
The scheme shows the gene construct WAP:6xHishGH encompassing rat *WAP* promoter (987 bp), histidine tag (6xHis), thrombin recognition site (Thr), and the entire genomic sequence of hGH (1,468 bp). The scheme also shows the locations of the primers used for the screening of the transgene. The distance between primers and codon start is shown in parentheses

Transgenic rabbit (No. 61) generation was as described previously (Lipiński et al. 2003). The founder male rabbit F0 was prepared by microinjection of a gene construct WAP:6xHishGH encompassing rat *WAP* promoter, histidine tag, thrombin recognition site, and the entire genomic sequence of hGH in pBSK + vector (Stratagene) into zygote (Scheme 1). When male No. 61 reached sexual maturity, his sperm was collected and used for the artificial insemination of non-transgenic females. As a result of this procedure, 32 transgenic rabbits of the F1 generation were generated. When transgenic females of the F1 generation reached sexual maturity, they were inseminated with the semen of the transgenic male to obtain homozygotes. As a result of this procedure, 34 transgenic rabbits of the F2 generation were generated (Table 1).

**Table 1:** Number of transgenic rabbits identified in the studied generations

Generation	Number of analyzed animals	Number of transgenic animals
F1 rabbits	109	32
F2 rabbits	49	34
Total	158	66

### Screening of transgene and sequencing

Total DNA was extracted from tissue ear bioptats using the guanidinium/isothiocyanate method as described by Ciulla et al. (1988). Two pairs of polymerase chain reaction (PCR) primers were placed on both sides of the junction between the promoter and the hGH gene encompassing part of the WAP promoter, 6 His tag sequence, thrombin recognition site, and the first hGH exon sequence. For the first screening, the primers WhGH2-F (5′-Cy5-AGTCTTCCTCCTGTGGGTC-3′) and WhGH2-R (5′-TCTCTCTCCATCCCTCCAG-3′) were used to amplify a 313-bp DNA fragment. In the second PCR screening, primers WhGH1-F (5′-Cy5-GTCCCAACCCAACCATTC-3′) and WhGH1-R (5′-TGGCGATACTCACATTCAGA-3′) were used to amplify a 524-bp DNA fragment. Primer locations are shown in Scheme 1. PCR was conducted in a Veriti Thermal Cycler (Applied Biosystems) in 25-μl reactions containing 125 ng total genomic DNA, 1× ReadyMix™ (Sigma Aldrich) and 0.125 μM of each primer PCR. The PCR amplification profile consisted of an initial denaturation at 94 °C for 5 min, followed by 30 cycles at 94 °C for 45 s, 55 °C for 45 s, 72 °C for 90 s, and a final extension at 72 °C for 10 min. PCR products were fractionated in 6 % polyacrylamide gel (19:1, AA:BA) under denaturing conditions on the ALFExpress sequencer. Two μl of PCR products were combined with the loading buffer and internal markers of 113 bp and 268 bp. For the analysis of results, Fragment Manager software (Pharmacia Biotech) was applied.

The nucleotide sequence of the whole transgene was performed on PCR products obtained by PCR with the genomic DNA as a template and the universal primers M13F (5′-CGCCAGGGTTTTCCCAGTCACGAC-3′) and M13R (5′-TCACACAGGAAACAGCTATGAC), both flanking the transgene region. Sequencing was conducted with automated genetic analyzers (Applied Biosystems Prism).

### Mapping of transgene

Mapping was performed by FISH using primary cell cultures of skin fibroblasts. Cells were cultured for 4 days in 37 °C in an atmosphere of 5 % CO_2_, DMEM, antibiotic, and 15 % fetal bovine serum (Sigma Aldrich). Metaphase plates were obtained by the addition of 0.05 μg/ml of colcemid. Cells were harvested with 0.1 % trypsin and 0.2 % EDTA, and treated with hypotonic solution and then fixed with a mixture of absolute methanol:glacial acetic acid (3:1). Analysis of the karyotype was performed using G-banding of metaphase chromosomes according to a routine procedure. The DNA probe specific for the transgene was labeled with biotin-dUTP by the Nick Translation Kit (Boehringer Mannheim) and purified on a Sephadex G-50 column. Hybridization was performed for 17 h at 37 °C. For signal detection, cell spreads were incubated with antibodies labeled with fluoresceine at 37 °C. Observation of a transgene signal was performed using a fluorescence microscope after standard staining of cells with DABCO/PI or DABCO/DAPI.

### mRNA expression level

For RNA analysis, exfoliated mammary epithelial cells were collected from 1 ml of milk from lactating transgenic females by centrifugation for 5 min at 500×g at 4 °C. From exfoliated mammary epithelial cells, total RNA was prepared with the RNeasy Mini RNA kit (Qiagen). Also, tissue biopsies (brain, heart, kidney, liver, and salivary gland) were collected and total RNA was isolated using the guanidinium/isothiocyanate/phenol/chloroform extraction (Chomczynski and Sacchi 1987). The detection of rhGH mRNA was carried out by reverse transcriptase reaction using the SuperScript®VILO™ cDNA Synthesis Kit. For PCR, 5 μl of cDNA solution were used. PCR was performed using 35 cycles (primer annealing 62 °C; extension 72 °C; denaturation 94 °C). The 173-bp cDNA fragment encompassing part of exon 3 and exon 4 was amplified using primer hGHcDNAF (5′-TTCATTCCTGCAGAACCCCCAG-3′) and primer hGHcDNAR2 (5′-TGTTGGCGAAGACACTCCTGAG-3′). cDNA coding sequences were sequenced bi-directionally using automated genetic analyzers (Applied Biosystems Prism). RNA isolated from the cells of non-transgenic animals was used in the same analysis as a control. In search of ectopic expression of the transgene, the studies were performed on the brain, heart, kidney, liver, and salivary gland.

### Immunoradiometric assay

Detection was performed in milk samples collected from transgenic and non-transgenic lactating females three times a week, once a day by turns with litter over a period of 2 weeks and stored at –80 °C until analysis. For analysis, samples of whole milk, defatted fraction, casein fraction, and whey fraction were separated. Eighty μl of each fraction were taken for immunoradiometric assay (IRMA, Polatom) analysis of rhGH concentration. Purification of growth hormone was performed from 8 ml of milk (4 °C), diluted with equal volume of phosphate buffer (50 mM phosphate buffer, 300 mM NaCl, pH 8.0). The sample was centrifuged at 2,000×g for 20 min at 4 °C. After removing a fat layer, urea was added up to a concentration of 8 M. Two ml of immobilized metal affinity chromatography resin Talon® (BD Biosciences) were equilibrated with phosphate buffer, pH 8.0. The fat-free milk sample was then homogenized on ice and mixed with Talon® resin. Proteins containing histidine tag were bound to resin during 16 h of incubation with gentle shaking at 4 °C. Unbound proteins were washed out with phosphate buffer, pH 7.0. Proteins containing histidine tag were eluted with phosphate buffer pH 5.0 and 4.0. Approximately 20 μl of each sample were used for 15 % denaturing gel electrophoresis (SDS-PAGE). For each test, small amounts of whole milk, defatted milk, unbound proteins, wash fractions, and elution fraction were used. Histidine tag was removed by thrombin cleavage of 20-μl samples by overnight incubation with 1 μl biotinylated thrombin, 5 μl thrombin cleavage buffer, and water added to a final volume of 50 μl. Following cleavage, thrombin was removed by binding to streptavidin agarose (16 μl settled resin per unit of enzyme). After 30 min of incubation at room temperature with gentle shaking, agarose was removed by centrifugation at 500×g for 5 min in a spin filter. Cleavage was monitored by gel electrophoresis of 10-μl aliquots.

### rhGH biological activity

The rhGH biological activity assay was based on a method described by Gout et al. (1980), with some changes (Ishikawa et al. 2000; Schulga et al. 2002). Nb211 cells (the rat growth hormone-dependent lymphoma cells) were grown in a complete medium RPMI 1640 containing 10 % fetal calf serum (FCS), 10 % horse serum (HS), 2 mM L-glutamine, 5 mM HEPES buffer, pH 7.4, penicillin (50 IU/ml ), streptomycin (50 μg/ml), and 50 μM of 2 β-mercaptoethanol in an atmosphere of 5 % CO_2_ at 37 °C. Cell viability was assured before each experiment by counting cells after trypan blue staining. Twenty μl of cell suspension were sampled with 20 μl of 0.4 % trypan blue solution, incubated for 5 min. Live and dead cells (stained blue) were counted by researchers manually under a microscope at 10× magnification in the Bürker chamber, in accordance with the two-sided principle in triplicate. After placing a cover glass on the edges close to the grating, a chamber was formed 0.1 mm deep, with 9 mm^2^ area and 9 mm^3^ volume. Before activity, bioassay cells were incubated overnight in a medium as described above with 1 % fetal calf serum instead of 10 % and without β-mercaptoethanol. The initial number of cells was 2.5 × 10^5^ live cells/ml. Two ml of cell suspension were transferred in tissue culture dishes. Samples with different concentrations of rhGH or hGH were added to culture dishes in triplicate. The number of live cells was calculated after 24, 48, and 72 h. The growth in control cultures (without GH) was essentially zero. The proliferation activity of recombinant human growth hormone was compared with commercially available human growth hormone expressed in HEK 293 cells (Sigma Aldrich).

## Results

The founder male No. 61 transmitted the transgene to the progeny and produced both transgenic females and males. Premature delivery of transgenic rabbits was not observed. All transgenic F1 animals were identified and milk samples of lactating females collected during lactation in the first pregnancy were analyzed for the presence of hGH. Further crossing of F1 heterozygotes generated male (No. 002) and female (No. 012) F2 homozygotes. Only one animal of F1 generation died after birth, but subsequent analysis showed no presence of transgene. All other animals had normal phenotype and showed no changes in their behavior.

**Fig. 1:**
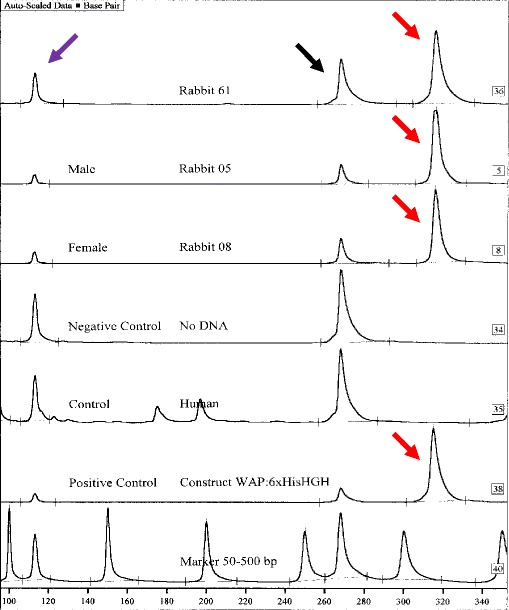
Transmission of rhGH transgene to rabbits of F1 generation. Presence of transgene was observed in both female and male heterozygote rabbits. A screening of the transgene was performed by polymerase chain reaction (PCR) encompassing 313-bp and 524-bp (not shown) DNA fragments. PCR primers were located upstream and downstream of histidine tag and thrombin recognition site of the gene construct. The *red arrows* indicate transgene in founder rabbit No. 61 and his F1 male and female offspring of the same size as the input DNA fragment (positive control in line 38). PCR observation was confirmed by sequencing. The *black arrow* indicates the internal marker of 268 bp. The *purple arrow* indicates the internal marker of 113 bp (Color figure online)

PCR analysis of DNA from ear samples from transgenic rabbits confirmed the presence of transgene of expected size. In the initial screening, two PCR reactions were performed, generating 313-bp and 524-bp DNA fragments. PCR products combined with internal markers were fractionated in polyacrylamide gel under denaturing conditions. No change in size of PCR screening fragments was observed in all transgenic animals of F1 generation. The screening data of the 313-bp fragment are presented in Fig. 1 (data for the 524-bp fragment not shown). A sequence analysis of the whole transgene confirmed that transgene in F1 animals was 100 % identical to a sequence of the gene construct used for the generation of founder animal No. 61.

**Fig. 2:**
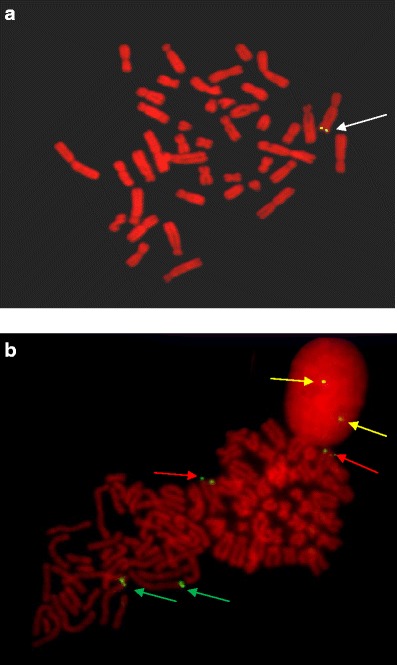
Mapping of a recombinant human growth hormone transgene was performed by fluorescence in situ hybridization (FISH) analysis using primary culture of skin fibroblasts (heterozygous and homozygous rabbits) and a transgenic gene construct as a molecular probe. The transgene was mapped to chromosome 7 on the metaphase plates of rabbits. **a** The *white arrow* indicates fluorescence signals observed in heterozygous female No. 08. **b** The *yellow arrows* indicate fluorescence signals observed in interphase nucleus, the *red arrows* the prophase, and the *green arrows* the metaphase of transgenic homozygote female rabbit number No. 012 of F2 generation (Color figure online)

Newborn F2 rabbits were tested for presence of the transgene by the same technology as that used for the screening of the F1 animals. The primary cell lines from skin fibroblasts of transgenic F1 and F2 rabbits were established and subjected to classical and molecular cytogenetic analysis. We discriminated heterozygous and homozygous animals on the basis of the FISH mapping. Examples of the results obtained for F1 heterozygous female rabbit (No. 08) and F2 homozygous female rabbit (No. 012) are shown in Fig. 2. The same results were observed for other transgenic heterozygotes and homozygotes. In all rabbits with integrated transgene confirmed by PCR screening, we detected the transgene in the q26-27 telomere region of chromosome 7 (the same location as that for founder animal No. 61). The location of the transgene is the same as that previously described in our paper, so we can deduce that integration was stable and it will not be lost during cell divisions.

**Fig. 3:**
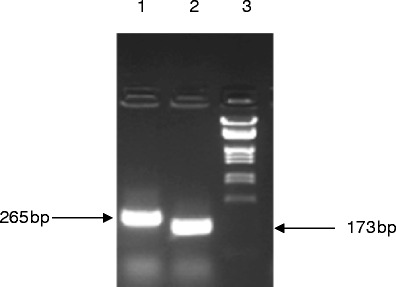
RT-PCR analysis of total RNA from exfoliated epithelial cells isolated from the milk of transgenic rabbits. Two μg of total RNA were subjected to reverse transcription using the primer located at the 3′ end of the rhGH gene. PCR was performed using primers located in exons 3 and 4. PCR of the cDNA sample resulted in an expected product of 173 bp, whereas genomic DNA yielded a 265-bp fragment. *Lane 1*, genomic DNA; *lane 2*, cDNA; *lane 3*, DNA size marker (λ DNA EcoRI/XbaI)

**Fig. 4:**
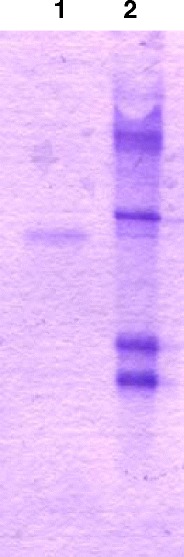
Electrophoresis of purified human growth hormone in 17 % polyacrylamide gel with SDS stained with Coomassie Blue. Electrophoresis showed the homogeneity of growth hormone. *Lane 1*, recombinant human growth hormone purified by affinity chromatography and cleaved with thrombin; *lane 2*, size marker, 12.3 kDa, 17.2 kDa, 30 kDa

Recombinant human growth hormone levels were determined in the serum of males and females, and in the milk of lactating females. Samples of serum and milk fractions were assayed using IRMA for estimation of the human growth hormone amount. The screening procedure for the presence of rhGH in the milk of rabbits was established with a sensitivity below 2 μIU/ml. Recombinant human growth hormone was detected in total unfractionated milk samples, in casein and whey fraction from transgenic females, but not in the serum samples of all transgenic animals. Milk fractions from wild type animals were negative for the presence of rhGH. The concentration of rhGH in milk samples observed for the transgenic homozygote rabbits was between 10 and 12 μg/ml (Table 2). rhGH levels in the milk of F1 heterozygous transgenic animals reached 0.5 μg/ml, with 97 % present in fat-free milk (casein fraction 53 %; whey fraction 44 %). The presence of rhGH mRNA was demonstrated in exfoliated mammary epithelial cells isolated from the milk during various lactation phases. Consistent evidence for the presence of rhGH RNA in mammary gland cells was obtained (Fig. 3). The transgene was not ectopically expressed in the brain, heart, kidney, liver, and salivary gland. All tested transgenic F1 females expressed rhGH-mRNA in exfoliated mammary epithelial cells collected from milk. As determined by the IRMA test, recombinant human growth hormone was observed in late fractions collected at pH 5.0 and early fractions eluted at pH 4.0 (Fig. 4). Fractions containing rhGH were pooled and digested by thrombin to remove histidine tag. An additional step of purification on a size exclusion column was needed to receive homogenous rhGH band during polyacrylamide gel electrophoresis under denaturing conditions (Fig. 4).

**Table 2:** Expression of recombinant human growth hormone in the milk of transgenic animals

Animals	Concentration of rhGH (μg/ml)
Serum of control rabbits	<0.0015
Serum of heterozygote rabbits	<0.0015
Serum of homozygote rabbits	<0.0015
Milk of control rabbits	<0.0001
Milk of heterozygote rabbits	0.02–0.512
Milk of homozygote rabbits	10–12

**Fig. 5:**
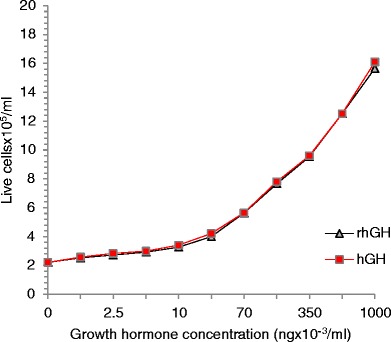
Growth hormone bioactivity assay. The effect of the biological activity of rhGH (*black line*) and human growth hormone (*red line*) measured by live cell numbers (cells × 10^5^/ml) after 72 h of growing under different growth hormone concentrations (ng × 10^-3^/ml) (Color figure online)

The activity of rhGH was determined by an increase in the amount of cells of the rat lymphoma line Nb211 in response to the rhGH purified product. Hormones devoid of lactogenic activity are not able to stimulate the proliferation of this type of cell line. The results of measurements of proliferative activity of rhGH and commercially available hGH after 72 h are shown in Fig. 5. The growth in control cultures (without GH) was essentially zero (2.57 × 10^5^). The results for rhGH purified product and commercially available human growth hormone do not differ from each other, so we can conclude that the biological activity of recombinant human growth hormone produced by transgenic animals corresponds with the activity of commercially available human growth hormone products.

## Discussion

The mammary gland is considered as the best available bioreactor. Extensive studies have shown that it can serve as a source of a variety of complex recombinant proteins (Houdebine 1994; Colman 1996; Clark 1998; Wall 1999; Jost et al. 1999; Houdebine 2000). In this report, we describe transgenic rabbits carrying the WAP:6xHishGH transgene with a checked expression recombinant human growth hormone in mammary glands at the transcription and translation levels, stability of the transgene transmission, and a mapped transgene by FISH. The normal pregnancy rates that were observed in this study demonstrated that the gene construct showed no interference with normal rabbit development. The results indicate that transgene WAP:6xHishGH became stably integrated into the host genome, can be transmitted to the offspring, and expressed. rhGH mRNA is specifically translated in the mammary gland and the product secreted into the milk as biologically inactive protein. Mammary epithelial cells, which are the source of milk-specific protein in the mammary gland, were confirmed to express the transgene.

There is a strong demand for recombinant growth hormone and its pharmaceutical applications has expanded during the last several years. The substantial cost limits dramatically the utilization of growth hormone. The primary reason for cost relates to the complexity of recombinant DNA technology and inherited costs of this technology. One rabbit female can produce up to 10 liters of milk per year. Assuming that expected expression is about 1–10 g/l, a rabbit colony of a few hundred animals can supply a substantial amount of hormone. We are to continue experiments of further crossbreeding the transgenic animals of the highest rhGH expression to obtain a representative group of animals to produce large amounts of recombinant protein. Alternative technologies to produce replacement therapeutic proteins, including growth hormone, are the subject of extensive research. During recent years, the production of a variety of human proteins in transgenic animals has been reported, including alpha 1-antitrypsin (Massoud et al. 1991), factor VIII (Niemann et al. 1999), anti-thrombin, human tissue type plasminogen activator (Ebert et al. 1991), protein C (Velander et al. 1992), interleukin 2, alpha-galactosidase, human thrombopoietin (Sohn et al. 1999), superoxide dismutase (Strömqvist et al. 1997), erythropoietin (Massoud et al. 1996), and lactoferrin (van Berkel et al. 2002), as well as growth hormone (Devinoy et al. 1994; Limonta et al. 1995; Dyck et al. 1999).

Generating homozygotes enabled a comparison of the expression of recombinant human growth hormone in the milk of transgenic animals in homozygous and heterozygous animals and to study the regulation of the expression. The expression can differ between animals and during lactation in one animal. It is an individual pattern. By crossbreeding animals with higher rhGH production, we can obtain animals with elevated levels of a recombinant protein. The breeding and biology of the reproduction of rabbits is relatively easy, so we can increase the level of production of rhGH protein by the generation of a greater number of lactating transgenic rabbits.

The integration of exogenous DNA into a single chromosomal site was observed. Confirmation that transgene was integrated into a single chromosomal site was performed by direct methods. The monitoring of transgene stability was observed through a breeding process and its transmission between generations.

Most mechanisms of stable gene transfer require chromosomal integration, which allows for the stable transmission of the transgene to all of the progeny. The mammalian genome usually forms silent and condensed heterochromatin, so a phenomenon called position effect manifests as the partial or complete loss of an expression. Telomeres are composed of short repeat sequences added onto the ends of chromosomes by telomerase, which forms a cap that serves multiple functions, including disguising the ends from appearing as double-strand breaks and preventing chromosome fusion. Telomeres can influence the expression of nearby genes by the effect called the telomere position effect (TPE) . The reversible silencing of genes near telomeres (TPE) has been extensively studied. Initial studies with mammalian cells failed to find evidence for TPE or for the repression of telomeric transgenes in a human/hamster hybrid cell line (Bayne et al. 1994). Another study found no apparent influence of a telomere length on the expression of an adjacent transgene in a human cell line (Sprung et al. 1996). Later studies, however, demonstrated TPE in human cancer cell lines by using transgenes located adjacent to telomeres, similar to the approach used with yeast, in which endogenous subtelomeric genes show variable expression levels (Fourel et al. 1999). In our case, with transgene mapped to the q26-27 telomere region of chromosome 7q, we did not observed a negative influence of the telomere position effect.

969 bp of the rat *WAP* promoter were sufficient to support a satisfactory level of expression of rhGH located at the telomere region of chromosome 7q. We assume that the fragment of *WAP* promoter used in our study included essential sequences required for directing expression exclusively to the mammary gland, since ectopic expression of the rhGH gene construct was not observed in this study, in spite of observations concerning the ectopic expression of other proteins under the *WAP* promoter (Wall et al. 1995; Barash et al. 1999).

Numerous reports are available on the expression of transgene in the mammary gland of transgenic rabbits (Limonta et al. 1995), but further experiments are desired regarding this issue. In this report, high and stable expression may reflect the positional effect of the transgene integration and, probably, multiple copy numbers of transgene. Transgenic rabbits generated in our studies could synthesize complex polypeptide in the mammary gland without showing any changes in the phenotype of secreting females and offspring, indicating that transgenic rabbits could serve as bioreactors.
